# Performance of Interleukin-6 and Interleukin-8 serum levels in pediatric oncology patients with neutropenia and fever for the assessment of low-risk

**DOI:** 10.1186/1471-2334-8-28

**Published:** 2008-03-06

**Authors:** Miriam Diepold, Peter Noellke, Ulrich Duffner, Udo Kontny, Reinhard Berner

**Affiliations:** 1 Department of Pediatric Oncology and Hematology, University Hospital of Bern, 3010 Bern, Switzerland; 2 Department of Pediatric Oncology and Hematology, University Hospital of Freiburg, Mathildenstrasse 1, 79106 Freiburg, Germany; 3 Department of Pediatrics and Adolescent Medicine, University Hospital of Freiburg, Mathildenstrasse 1, 79106 Freiburg, Germany

## Abstract

**Background:**

Patients with chemotherapy-related neutropenia and fever are usually hospitalized and treated on empirical intravenous broad-spectrum antibiotic regimens. Early diagnosis of sepsis in children with febrile neutropenia remains difficult due to non-specific clinical and laboratory signs of infection. We aimed to analyze whether IL-6 and IL-8 could define a group of patients at low risk of septicemia.

**Methods:**

A prospective study was performed to assess the potential value of IL-6, IL-8 and C-reactive protein serum levels to predict severe bacterial infection or bacteremia in febrile neutropenic children with cancer during chemotherapy. Statistical test used: Friedman test, Wilcoxon-Test, Kruskal-Wallis H test, Mann-Whitney U-Test and Receiver Operating Characteristics.

**Results:**

The analysis of cytokine levels measured at the onset of fever indicated that IL-6 and IL-8 are useful to define a possible group of patients with low risk of sepsis. In predicting bacteremia or severe bacterial infection, IL-6 was the best predictor with the optimum IL-6 cut-off level of 42 pg/ml showing a high sensitivity (90%) and specificity (85%).

**Conclusion:**

These findings may have clinical implications for risk-based antimicrobial treatment strategies.

## Background

Infections are still the major cause of treatment-related morbidity and mortality in cancer patients [1]. The malignant disease and the intensive chemotherapy may cause an impaired host defence to infection. Key factors are the intensity and duration of neutropenia, but a decreased function of granulocytes and disturbances of natural barriers may substantially add to the risk of serious infections [2]. Patients with chemotherapy-related neutropenia and fever are usually hospitalized and treated on empirical intravenous broad-spectrum antibiotic regimens until the patient is afebrile, the blood cultures are negative and the absolute neutrophile count (ANC) has recovered to > 500/μl [3]. Patients without a documented clinical focus of infection and without microbiological evidence for a causative organism usually have a short duration of fever and low risk of developing clinical complications. Unfortunately, culture results become available only after 2 or 3 days and clinical information at fever onset that can be used to establish prediction rules lacks sensitivity and specificity. There are a number of studies that have evaluated diverse markers of inflammation as predictors of patients subgroups with different types of infection [4-6]. If there were parameters that could define a group of patients with a low risk of sepsis, simplified approaches may include early discharge from the hospital, intravenous treatment as outpatients, or even the use of oral antimicrobial therapy. This should reduce the risk of nosocomial infection and development of resistant bacteria. Other advantages would be cost savings and an improved quality of life for these patients.

The inflammatory response reflects an ongoing collaboration between tissue macrophages and mast cells, vascular endothelial cells and circulating phagocytes. T-cells, B-cells, natural killer cells and platelets are also involved in the inflammatory response. The release of soluble inflammatory mediators plays a crucial role in activating and coordinating this process. The proinflammatory cytokines Tumor Necrosis Factor (TNF)-α and Interleukin (IL)-1 have a broad range of activities in the acute inflammatory response. IL-6 is an extremely pleiotropic cytokine with important effects on the growth and differentiation of T and B cells, on the induction of the hepatic acute phase response and enhancement of proliferation of hematopoietic progenitor cells. IL-6 synthesis and secretion is stimulated by IL-1. IL-8 is released from monocytes, endothelial cells, neutrophils and many other cells in response to IL-1 and TNF-α and activates neutrophils, T cells and basophils. [7,8].

The aim of this study was to determine the value of serum levels of IL-6, IL-8 and C-reactive protein (CRP) as predictors for sepsis or prolonged fever in children with fever and neutropenia due to chemotherapy at the start of a febrile episode. We aimed to analyze whether IL-6 and IL-8 could define a group of patients at low risk of septicemia. This might lead to the identification of patients in the future who can be discharged earlier from the hospital, or even treated under outpatient conditions.

## Methods

### Patients

The study was performed at the Department of Pediatric Oncology and Hematology at the University Hospital Freiburg, Germany. Sixty-nine patients with cancer or haemotological disease with febrile neutropenia were included in the study. Approval by an ethics committee was not necessary at the time of the study. Informed consent was given by the parents or patients. Their characteristics are depicted in table 1.

**Table 1:** Patients characteristics

**number of patients (n)**	69
**sex (m/w)**	42/27
age at diagnosis (median, range)	7 8/12 yrs. (1 month – 20 years)
	
**leukemia (n)**	25
ALL	21
AML	1
JMML	1
AML-Relapse, after SCT	2
	
**solid tumors (n)**	39
brain tumor	5
bone tumor	4
Hodgkin's disease	3
NHL	10
others	17
	
**hematological disorders (n)**	5
after SCT	4
no SCT	1

ALL: acute lymphatic leukemia; AML: acute myeloid leukemia; SCT: stem cell transplantation; NHL: Non-Hodgkin's-lymphoma.

Patients in good clinical condition and an expected duration of aplasia of less than five days were initially treated with Ceftriaxone (80 mg/kg body weight/day). Patients in poor clinical condition or an expected duration of aplasia of five days or longer were treated with Ceftazidime (150 mg/kg body weight/day). Likewise, patients with acute myeloid leukemia, high risk lymphatic leukemia, B-non-Hodgkin's lymphoma, relapse of acute leukemia, or patients undergoing bone marrow transplantation were treated with Ceftazidime.

All together, 141 febrile episodes (defined as fever > 38.5°C once, or 38°C > 1 h) of 69 patients were analysed. "Episode" was defined as the time between the first blood sample and the last blood sample taken on the day on which antibiotics were stopped. All patients were neutropenic at the onset of fever. Neutropenia was defined as absolute neutrophil count below 0.5 × 10^9 ^/l. The blood samples were taken within 24 hours since the start of fever and than daily. The data from 123 of the 141 episodes enrolled in the study could be analyzed. In 18 of the episodes, the first blood sample could not be taken within 24 hours since the start of fever. These episodes were excluded from analysis. Nearly 75% of the patients experienced one episode, two patients six episodes. The duration of an episode ranged between one and 95 days, the median duration was six days.

Three separate groups of febrile episodes were defined (table 2). It was supposed that patients with a febrile episode up to three days without a positive blood culture and without clinical signs of shock or a mirobiologically documented local infection had another cause of fever than sepsis, for example fever due to chemotherapy or a viral infection. Patients with a positive blood culture result were classified belonging to the septic group (group is called episep). Patients with a febrile episode of five days or more were assigned to a separate group (group is called epi5). On the basis of the long duration of the episode we supposed that these patients had either a serious infection or signs of clinical sepsis without microbiologically documented infection. Due to this group definition, there were 10 episodes that could not be classified to one of the groups (duration of the episode four days with negative blood culture). These episodes were not taken into consideration while testing the differences between the groups.

**Table 2:** Description of the different groups of episode

**Group**	**Description**	**n**	**duration of episode in days; median (range)**
Epi3	duration of episode ≤ 3 days, blood culture negative	28	2 (1–3)
Epi5	duration of episode ≥ 5 days, blood culture negative	71	8 (5–95)
Episep	documented Gram-negative or Gram-positive blood culture	14	10 (3–36)
Intermediate group (not included in the analysis)	duration of episode 4 days, blood culture negative	10	4

### Laboratory analysis

All patients were examined daily for clinical signs of infection. Prior to antibiotic therapy blood cultures, cultures from urine and suspected lesions were taken. Additional blood cultures were taken during the study period according to clinical signs. During the febrile episodes complete white blood counts (WBC), differentials, CRP, IL-6 and IL-8 were determined daily. Cytokine concentrations in the serum were measured by a fully automated random access system (Immulite^®^) which allows the immediate individual analysis of any blood sample at any time.

### Statistical analysis

Nonparametric bivariate statistics were used for testing of the association between variables. The Friedman test was used to compare median-values of 3 or more groups in related samples [9]. If this global-test was statistically significant a pairwise post hoc test (Wilcoxon-Test) was performed [10].

For unrelated samples the Kruskal-Wallis H test was used to test the differences in the median-values of more than 2 groups (post hoc test: Mann-Whitney U-Test) [11,12]. P values less than 0.01 were considered to indicate statistical significance. To determine the cut-off-level with optimal sensitivity and specificity Receiver Operating Characteristics (ROC) were calculated [13]. Statistical analysis was performed using SPSS for Windows 11.0.1 (SPSS Inc, Chicago, IL).

## Results

**Figure 1:**
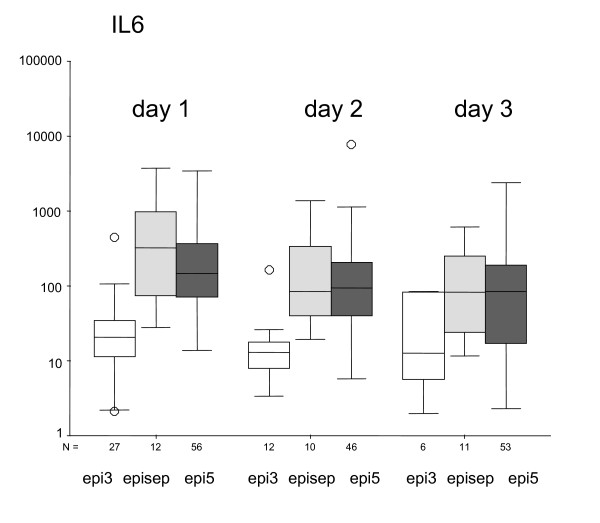
**IL-6 (pg/ml) according to the different groups of episode at the days of fever 1, 2 and 3**. The median is marked by the center horizontal line of the central box. The lower and upper hinges comprise the edges of the central box representing the interquartile range. The Hspread is the absolute value of the differences between the values of the two hinges. The whiskers (⊥) show the range of values which fall within 1.5 Hspreads of the hinges. Values outside the inner fences (+/- 1.5 Hspread) are plotted with empty circles (O).

All together, 141 febrile episodes (defined as fever > 38.5°C once, or 38°C > 1 h) of 69 patients were analysed. The data from 123 of the 141 episodes enrolled in the study could be analyzed. In 18 of the episodes, the first blood sample could not be taken within 24 hours since the start of fever. These episodes were excluded from analysis. In Fig. 1 the comparison of IL-6 between the defined groups on the first three days of fever is given.

**Figure 2:**
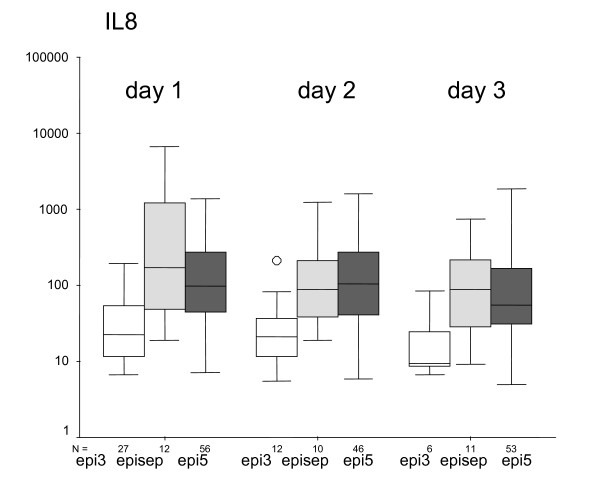
IL-8 (pg/ml) according to the different groups of episode at the days of fever 1, 2 and 3.

**Figure 3:**
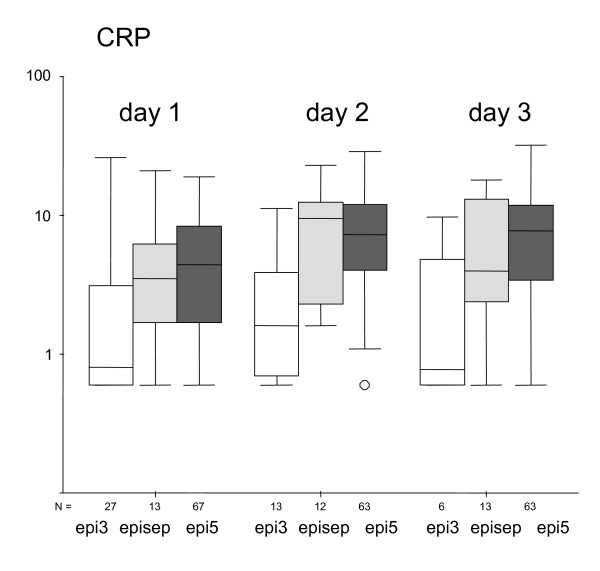
CRP (mg/dl) according to the different groups of episode at the days of fever 1, 2 and 3.

On day 1, IL-6 is significant lower in group epi3 (median 21 pg/ml) than in group epi5 (median 146 pg/ml) or group episep (median 326 pg/ml; p < 0.01). This difference exists also on day 2. On day 3, there is no difference between the groups. There is also no statistically difference between groups epi5 and episep. In Fig. 2 the comparison of IL-8 between the defined groups on the first three days of fever is given. On day 1, IL-8 is significant lower in group epi3 (median 22 pg/ml) than in group epi5 (median 97 pg/ml), and group episep (median 175 pg/ml; p < 0.01). This difference also exists on day 2. On day 3, only a tendency can be observed (p = 0.01). There is no statistically significant difference for IL-8 between groups epi5 and episep. In Fig. 3 the comparison of CRP between the defined groups on the first three days of fever is given. On day 1, CRP is significantly lower in group epi3 (median 0.8 mg/dl) than in group epi5 (median 4.4 mg/dl) and group episep (median 3.5 mg/dl; p < 0.01). This difference can also be seen on day 2, but not on day 3.

Similarly, the maximum values of IL-6, IL-8 and CRP differ significantly between the groups. The values in group epi3 (IL-6 21 pg/ml; IL-8 22 pg/ml, and CRP 0.8 mg/dl) are significantly lower than in the two other groups (p < 0.01). Between the groups episep and epi5 no difference is observed (episep: IL-6 569 pg/ml, IL-8 175 pg/ml, CRP 10.4 mg/dl; epi5: IL-6 268 pg/ml, IL-8 246 pg/ml, CRP 11 mg/dl).

To test which cut-off-level for IL-6 or IL8 would be useful to seperate fever of unknown origin from sepsis, first the group of children with documented sepsis is compared to the two other groups (epi3 und epi5). From the ROC curve, a cut-off value of 240 ng/l is calculated for IL6, resulting in a sensitivity of 67%, a specificity of 75%, and a positive predictive value (PPV) of 28%. For IL-8 a cut-off level of 90 ng/l is found with a sensitivity of 67%, a specificity of 62%, and a PPV of 20%. Because of the low values for sensitivity und specificity, no clear separation between the two groups can be made.

In the following analysis the group of children with sepsis and the group of children with a longer episode of fever is combined (episep + epi5), and compared to the group of children with low-grade fever (epi3). In this analysis, a cut-off value of 42 pg/ml for IL6 is found, resulting in a sensitivity of 90%, a specificity of 85%, and a PPV of 94%, meaning that in 94% of the cases patients with an IL6-value at the first day of fever above the cut-off-level 42 pg/ml will develop sepsis or a longer episode of fever. The NPV (negative predictive value) is 77%, meaning that in 77% of the cases patients with an IL6-value at the first day of fever below the cut-off-level will not develop sepsis or a longer fever period.

Thus, measuring IL6 at the first day of fever allows to identify a group of patients whith a high risk to develop sepsis or a prolonged episode of fever. On the other hand, patients with IL6-values below 42 pg/ml on the first day of fever have a high chance to belong to a group of patients with a short episode of fever.

For IL-8 and CRP the values for sensitivity, specificity and PPV were less impressive. For IL-8 a cut-off of 30 pg/ml results in a sensitivity of 87%, a specificity of 59%, and a PPV of 84%. For CRP a cut-off of 1 mg/dl was associated with a sensitivity of 83%, a specificity of 59%, and a PPV of 86%, respectively.

## Discussion

In this study we analyzed the significance of serum levels of the proinflammatory cytokines IL-6 and IL-8 as well as C-reactive protein as predictors for severe bacterial infection or bacterial sepsis in children with fever and neutropenia during cancer chemotherapy. We applied a new method for the measurement of cytokines, a fully automated chemiluminescence immunoassay, which allows the immediate random access analysis of blood samples.

Previous studies have established the value of cytokines such as IL-6 and IL-8 to predict bacterial infection in the neonate with high sensitivity and specificity [14]. Other studies have used similar criteria, affirming their predictive value in the setting of fever and neutropenia among patients during cancer chemotherapy. De Bont et al. showed that plasma levels of IL-6 and IL-8 can be used to define a group with low risk of septicaemia among cancer patients, aged between 1 and 66 years, with fever and neutropenia [15]. Data reported by Lehrnbecher et al. showed the potential usefulness of IL-6 and IL-8 as early indicators for life-threatening infections in febrile cancer patients with neutropenia [16].

In this study, it could also be shown that plasma IL-6 and IL-8 levels can possibly be used to define a group with low risk of septicaemia among children with fever and neutropenia. The sensitivity and specificity of each parameter at different cut-off levels were analyzed to predict bacteremia or severe bacterial infection among all febrile episodes at the time of admission. In order to define a group with low risk of septicaemia, three separate groups of febrile episodes were defined (Tab. 2). It was supposed that patients with a febrile episode up to three days without a positive blood culture and without clinical signs of shock or a mirobiologically documented local infection had another cause of fever than sepsis, for example fever due to chemotherapy or a viral infection. Patients with a positive blood culture result were classified belonging to the septic group. We chose the time period of 3 and 5 days, respectively, because this is usually the time point where the decision has to be made if antibiotics have to be kept, changed or withdrawn. In our experience episodes of up to 3 (and 5, respectively) days of antibiotics are very unlikely to be associated with true septicemia or invasive bacterial infection. Therefore, it was our intention to define a group of patients with a probably low risk of severe infection. There is a grey area between the low and the high risk group. In order to achieve a high specificity of the low-risk definition, this postulated grey area was included into the high-risk group, and the cut-off level was set accordingly.

In predicting bacteremia or severe bacterial infection, the optimum IL-6 cut-off level was 42 pg/ml with a high sensitivity (90%) and specificity (85%), the positive predictive value was 94%. IL-6 was the best predictor, the other parameters showed a lower sensitivity and specificity. The optimum IL-8 cut-off level was 30 pg/ml with a high sensitivity (87%) and a lower specificity (59%). The optimum CRP cut-off level was 1 mg/dl with a sensitivity of 83% and a specificity of 59%. The group of patients with loy-grade fever and a documented gram positive or gram negative blood culture are small (28 and 14 patients respectively), a larger group would e.g. reveal IL-8 to be the most accurate parameter.

Lehrnbecher et al. found that either IL-6 or IL-8 might be useful parameters in a febrile child with cancer and neutropenia at the time of admission [16]. IL-6 and IL-8 levels were higher in patients with either bacteremia due to Gram-negative organisms or fungal infections than in patients with febrile episodes without an identifiable source. De Bont et al. also found that IL-6 and IL-8 were highly correlated [15]. Addition of IL-6 to a model containing IL-8, or vice versa, did not significantly improve the fit. Engel et al. compared serum levels of procalcitonin with IL-8, and showed that IL-8 was more sensitive and specific than procalcitonin in the prediction of Gram-negative bacteremia [17].

In contrast, Fleischhack et al. suggested that procalcitonin is a more useful diagnostic parameter in febrile cancer patients than IL-6, IL-8 and CRP [18]. Strychjewski et al. combined calcitonin precursors with interleukin-8 as a marker of bacterial sepsis in febrile, neutropenic children. IL-8 was increased in septic children compared with those without bacterial sepsis but there were no significant differences in the values of IL-6 between septic and nonseptic patients. Using CTpr at 24 hrs in addition to interleukin-8 at 48 hrs produced the best-fit models associated with sepsis [19]. Kitanovski et al. suggested that IL-6 and PCT are more sensitive and specific early markers of bacteremia/clinical sepsis than CRP in children with febrile neutropenia. Sequential determinations improved the diagnostic accuracy of PCT, but not of IL-6 [20].

Hodge et al. simultaneously determined multiple cytokines in childhood oncology patients with febrile neutropenia and found increased IL-8 or IL-5 correlating with culture-positive infection [21]. Nijhuis et al. used a risk assessment model combining clinical parameters and plasma interleukin 8 levels to define 3 risk groups among outpatients with febrile neutropenia (children and adults). Patients at low risk for bacterial infection did not receive antibiotics [22].

Serum levels of CRP are commonly used to assess and monitor the acute-phase response. Several studies found a high sensitivity of serial CRP measurements but a low specificity, as it was shown also in this study [4,16,23,24].

Hospital treatment of all patients with neutropenia with empirical intravenous broad-spectrum antibiotic regimens at the first signs of fever has drastically reduced morbidity and nearly eliminated mortality. For high-risk patients, this treatment is appropriate considering the possibility of rapid deterioration. But nearly two-thirds of all children are treated without having a source of the fever identified [25,26]. Antibiotic therapy is given for at least 3 days or even longer, which results in the risk of exposure to nosocomial pathogens and selection for resistant bacteria or even fungal infections. For these patients it would be of great interest to have a reliable diagnostic marker to identify them belonging to a low-risk group on day one of a fever episode. Studies that used factors like IL-6 or IL-8 to define a low risk group were often determined after three days but there are also studies (e.g. Oude Nijhuis et al) that determined a low risk group after 12 hours and studies that determined a low risk group within 24 hours when using clinical parameters (e.g. Aquino et al, Santolaya et al).

The factors used so far to define a low-risk group were absolute neutrophil count, the absence of comorbidity such as hypotension and respiratory compromise, the duration of fever (at least 24 h afebrile), the type of malignancy and disease status were the most important indicators [27].

Studies published by Kern et al. and Freifeld et al. concerning adult patients determined oral versus intravenous empirical antimicrobial therapy for low-risk febrile patients with neutropenia during cancer therapy [28,29]. The criteria to identify low-risk patients were the following: they excluded patients who had received allogeneic bone marrow or peripheral-blood stem-cell transplants, those with acute leukemia, those in whom granulocytopenia was expected to last longer than 10 days and those with shock or any other condition that required intravenous supportive therapy or precluded oral intake of drugs. They showed that oral therapy with ciprofloxacin plus amoxicillin-clavulanate was as effective and safe as intravenous therapy. Further carefully designed studies are needed to specify the conditions under which outpatient therapy will be an acceptable choice. In any case, the establishment of careful rules with each patient and family is essential.

## Conclusion

In the present study, plasma levels of IL-6 and IL-8 allowed to define a group with short duration of the fever episode and a group with severe infection or even blood culture positive sepsis. IL-6 was the best parameter. Further studies are needed to answer the question whether the observed results are of clinical relevance and might be used for early discharge of a selected group of patients, an outpatient therapy with antibiotics given once daily intravenously (e. g. Ceftriaxone), or even the use of oral antimicrobial therapy. This may ultimately result in substantial cost savings, reduced exposure to nosocomial pathogens, it may improve the quality of life of the children, and reduce the disruption of family life.

## Competing interests

The author(s) declare that they have no competing interests. There was no financial support of the study.

## Authors' contributions

MD participated in the conception and the design of the study, was responsible for the coordination and the acquisition of data, participated in the interpretation of data and drafted the manuscript. PN participated in the conception and the design of the study, performed the statistical analysis and participated in the interpretation of data. UD participated in the acquisition and the interpretation of data. UK participated in the acquisition and the interpretation of data. RB was responsible for the conception and the design of the study, and responsible for the data interpretation. All authors critically revised the draft of the manuscript and read and approved the final manuscript.

## Pre-publication history

The pre-publication history for this paper can be accessed here:


